# Feeding Ecology of the Critically Endangered *Gobio insuyanus* (Gobionidae)

**DOI:** 10.1002/ece3.71156

**Published:** 2025-03-20

**Authors:** Julian E. Johnson, Baran Yoğurtçuoğlu, Şerife Gülsün Kırankaya, Fitnat Güler Ekmekçi

**Affiliations:** ^1^ Department of Biology Faculty of Sciences, Hacettepe University, Beytepe Campus Ankara Türkiye; ^2^ Department of Biology Faculty of Art and Sciences, Düzce University, Konuralp Campus Düzce Türkiye

**Keywords:** Central Anatolia, conservation, diet composition, index of relative importance, stomach content

## Abstract

Despite the critical conservation status of the endemic gudgeon *Gobio insuyanus*, its feeding ecology remained unstudied. This research addresses this gap by investigating the diet of *G. insuyanus* in the Insuyu spring–stream system of Central Anatolia (Turkey) analyzing spatial, temporal, and intraspecific variations. We compared two distinct habitats: a stable spring and its continuum, a fluctuating stream. Results indicate that *G. insuyanus* is an omnivore, consuming primarily detritus, Gammarids, and Diptera larvae. Feeding intensity was higher in the spring habitat, particularly in summer, possibly related to its stable temperature and lower turbidity. Dietary diversity was higher in the stream, potentially reflecting its greater habitat complexity, but decreased in both habitats in autumn, suggesting a seasonal decline in prey availability. A significant ontogenetic shift in diet was observed; mature individuals exhibited a narrower niche and preferred larger prey, probably due to increased gape size, improved foraging ability, and higher energy requirements. No significant dietary differences were found between the sexes. The results have conservation implications, emphasizing the need to maintain the integrity of both habitats. Future research incorporating fish movement data with feeding ecology will further improve our understanding and inform more targeted conservation strategies.

## Graphical abstract

This study investigates the diet of the critically endangered *Gobio insuyanus* in the Insuyu spring–stream system, revealing omnivorous feeding habits with a preference for detritus, Gammarids, and Diptera larvae. Ontogenetic dietary shifts were observed, with mature individuals specializing in larger prey, while habitat and seasonal variations influenced feeding activity and dietary diversity. These findings highlight the ecological importance of both habitats and emphasize the need for conservation efforts to preserve this endemic species.

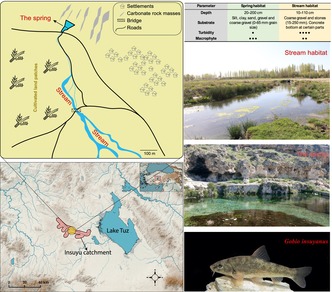

## 1 Introduction

Freshwater ecosystems are recognized as significant biodiversity hotspots on a global scale, despite covering less than 1% of the Earth's surface (Strayer and Dudgeon 2010; Tockner 2021). However, the resilience of these ecosystems is currently at a critical threshold, with evidence suggesting that freshwater biodiversity is declining at a faster rate than terrestrial ecosystems (Ricciardi and Rasmussen 1999; Sala et al. 2000). These systems face numerous threats, including habitat fragmentation, pollution, climate change, and especially water abstraction for agricultural and other purposes (Tickner et al. 2020; Tockner 2021). Land use changes and landscape alterations also significantly contribute to the degradation of freshwater basins, impacting fish functional diversity (Seabra et al. 2023). This situation is particularly severe in Central Anatolia (Turkey), where a semi‐arid climate combined with factors such as habitat fragmentation, water abstraction, and pollution are seriously threatening range‐restricted endemic fish species (Darwall et al. 2015; Yılmaz et al. 2021). Climate projections suggest that half of these endemics could face significant losses in their ecologically suitable habitats; in some cases, a complete loss of viable habitats is expected by 2081–2100 under high‐emission climate scenarios (Korkmaz et al. 2023).

Cihanbeyli Gudgeon (*Gobio insuyanus*), one of these endemic species, is critically endangered due to being restricted to a single small karst spring and its downstream reach in the Insuyu stream in the western Lake Tuz basin (Freyhof et al. 2025; Smith et al. 2014). This subbasin of Central Anatolia has been identified as a Key Biodiversity Area (KBA) and proposed as an Alliance for Zero Extinction (AZE) site due to its global significance as it holds the entire global population of *G. insuyanus* (Darwall et al. 2015). The area is also home to five other narrowly endemic fish species threatened severely by ongoing habitat loss (Darwall et al. 2015; Freyhof et al. 2025). Excessive water abstraction for agricultural purposes, in addition to the implementation of regulations on water levels, poses a significant challenge, as it directly results in a reduction in the availability of suitable habitats and leads to fragmentation (Darwall et al. 2015). Consequently, fish populations that survive within fragmented habitat patches serve as vital conservation units, on which management strategies should be specifically designed. In this context, it becomes essential to investigate the ecology of fish inhabiting these habitats in order to develop effective and targeted conservation measures.

Despite its critical status, *G. insuyanus* remains largely under‐studied. Studies on some congeners—such as *Gobio gymnostethus* (Melendiz Stream) (Özdemir 2012) and *G. hettitorum* (Yeşildere Stream) (Özdemir and Erkakan 2012)—have been examined for their growth and reproductive traits. Feeding and growth studies on *Gobio bulgaricus*, from the Maritsa River westward to the Struma, also provide valuable comparative information about the genus (Saç and Özuluğ 2020). However, data on *G. insuyanus* is mostly limited to its formal description (Ladiges 1960) and its length‐weight relationship analyses (Ergönül et al. 2019; Erk'akan et al. 2014; Şenyi̇ği̇t and Mazlum 2022). This knowledge gap is of particular concern given the limited distribution of the species and the distinct hydrological characteristics of its unique habitat, which is a spring‐stream continuum. In such an environment, potential microhabitat variations in life history or feeding behavior could potentially influence the ability to withstand habitat degradation. For instance, the question of whether individuals migrate between the spring and the stream or exhibit site‐specific adaptations for feeding remains unanswered, resulting in a knowledge deficit that prevents the development of targeted conservation planning. This is also indicated by the observation that the physical and chemical properties exhibit significant differences (e.g., stable vs. fluctuating or turbid vs. clear) between the stream and spring habitat, which may potentially influence the feeding strategies and habitat use of the species.

This study, therefore, addresses these critical gaps by presenting the first comprehensive analysis of the feeding ecology of *G. insuyanus*. The present study considers spatial, temporal, and intraspecific variation in dietary composition and feeding strategies. In addition, it assesses whether habitat loss may influence the ecological stability of the species. Consequently, it assesses the potential fine‐scale differences in feeding ecology between spring‐ and stream‐dwelling individuals, between seasons, and between fish at different maturity stages. Understanding this variation is essential to assess the adaptability of the species to environmental change and to inform targeted conservation strategies to mitigate the effects of habitat degradation.

## 2 Materials and Methods

### 2.1 Permissions and Compliance With Ethical Standards

Fish collections were approved and granted by the Ministry of Food, Agriculture, and Livestock, General Directorate of Fisheries and Aquaculture (Codes for the protocols: B.12.0.KKG.0.17/106.01–11‐01 and 76,000,869/150–4199). All applicable international, national, and/or institutional guidelines for the care and use of animals were followed.

## 3 Site Description and Data Collection

**FIGURE 1:**
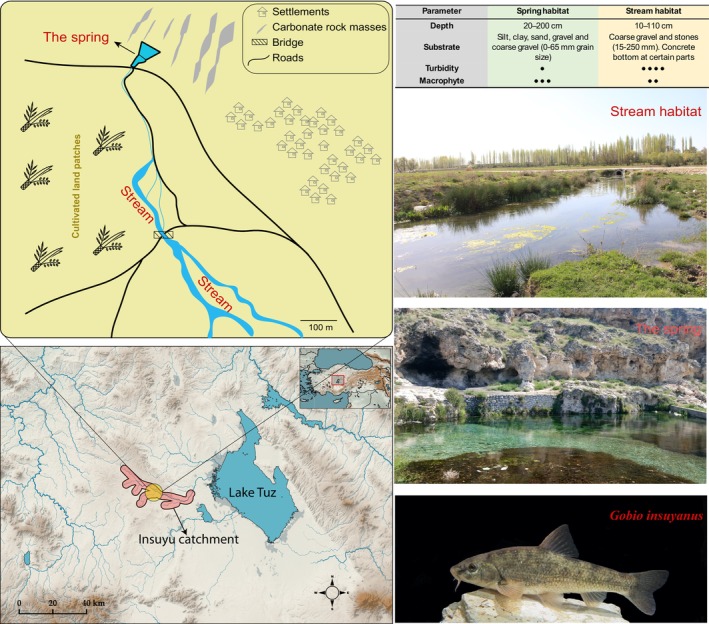
Map and habitat characteristics of the study area in the Insuyu Catchment. (Upper‐left) Schematic map showing the location of the spring and connected stream habitats, with surrounding land use features. (Upper‐right) Summary of key habitat parameters in spring and stream habitats. (Middle‐right) Photographs of the stream habitat (top) and spring habitat (bottom). (Lower‐left) Regional map displaying the location of the Insuyu Catchment in central Anatolia. (Lower‐right) Photograph of *Gobio insuyanus*. For turbidity and macrophyte density; ● very low, ●●low, ●●●high, ●●●●very high.

The study was conducted in the Insuyu Stream, an aquifer‐fed water system located within the İnözü Valley in Cihanbeyli district (Central Anatolia). Geographically, the Insuyu Stream originates near the village of Pınarbaşı and flows westward towards Tuz Gölü (Salt Lake) (Figure 1). However, recent field observations and satellite imagery indicate that the stream does not reach the lake. This is probably due to over‐extraction of groundwater for intensive agriculture, reduced rainfall, and some impoundments that have significantly altered the natural flow regime. The topography of the Inözü Valley, which extends in an east–west direction, is distinguished by the presence of karstic landforms (Bozdağ and Göçmez 2013). The region is subject to a continental climate, characterized by arid and hot summers, and exhibits high evaporation rates. It is located within a particularly arid area of Turkey, where minimal annual rainfall is observed. Consequently, this combination of factors significantly increases the vulnerability of its hydrological systems (DKM 2019). Insuyu is the only known range for *Gobio insuyanus*, which is found in only two consecutive habitats: the main aquifer outlet and its ongoing stream, both of which cover an area of only approximately 50 km^2^. As illustrated and summarized in Figure 1, the main spring outlet is characterized by a relatively deeper, stagnant water body with depths ranging from about 20 to 200 cm, while the stream has a slow flowing habitat with depths ranging from about 10 to 110 cm. The water temperature in the spring habitat is typically stable, ranging from 13.5°C to 15.5°C throughout the year. In contrast, the water temperature in the stream habitat exhibits seasonal variability, ranging from 9.7°C in winter to 21.5°C in the summer period. The substrate composition also differs between these habitats; the spring area is dominated by coarse gravel and stones sized between 15.0 and 250.0 mm, with sections of concrete substrate also present. By contrast, the stream habitat contains a mixture of silt, clay, and sand, gravel, with grain sizes ranging from 0.1 to 65.0 mm. Overall, the physical and chemical properties of water exhibited a significantly higher stability in the spring habitat while demonstrating seasonal fluctuations in the stream habitat. Furthermore, the stream habitat is subject to direct exposure to the nutrient load of surface waters, as it flows among cultivated lands. Conversely, the spring habitat is situated on a karstic bed, where the groundwater emerges, resulting in a comparatively lower level of turbidity and nutrient load. Habitat loss in this area is the key concern, driven by groundwater overuse that has led to critical water level declines (DKM 2019). Consequently, both the spring and the associated stream are experiencing significant environmental stress and are at risk of irreversible loss soon. The map of the study site and pictures of sampling habitats are also given in Figure 1.

The fish were collected using a backpack electrofishing device (Samus 725 MS) with two practitioners, as well as a hand‐held dip‐net with a 1 mm mesh size. Sampling was conducted in the same 100 m stretch of the stream and the same ~100 m^2^ area of the spring habitat during each survey to ensure temporal consistency and allow comparative assessments across sampling events. The fishing effort covered all available microhabitats (e.g., riffle, glide and pools) within this stretch, extending up to ~1 m from the terrestrial edge of the riverbank in the wadable parts. In the spring habitat, a beach seine (1.5 m × 1.5 m 3.0 m) with a mesh size of 4 mm was used in addition to electrofishing. The sampling was conducted on a monthly basis over a period of 10 months, from June 2018 to September 2019, ensuring that the annual seasonal cycle was covered.

### 3.1 Laboratory Procedure

Immediately after collection, fish samples were preserved in 10% formalin on site and then transported to the laboratory for further analysis. In the laboratory, the total (TL) and fork length (FL) of each fish was measured to the nearest 0.01 mm using a digital caliper, and both total and eviscerated weights were recorded to the nearest 0.001 g using a digital balance. Sex was determined by examination of the gonadal structures under a stereomicroscope. The stomachs of fish were eviscerated, and the contents removed and weighed. An initial macroscopic examination was carried out to determine the presence of larger prey items. For a more detailed assessment, the stomach contents were further analyzed using an Olympus SZ‐X12 stereomicroscope. Prey items were quantified using a 1 mL Sedgewick Rafter counting chamber. The volume of each prey organism was estimated either by compressing the food content into a calculable area or by approximating the volume based on the closest geometric shape of the organism (Hellawell and Abel 2006; Sun and Liu 2003). Shape measurements were performed using FIJI Image J software (Schindelin et al. 2012). Identifiable parts of organisms, such as heads, were counted as individual prey items and identified to the lowest taxonomic level possible. Clumped debris was categorized as detritus and treated as uncounted prey item.

## 4 Data Analysis

To assess feeding intensity, the Fullness Index (FI) was calculated using the following equation: FI = (stomach content weight/total fish body weight) × 10,000. The hypothesis that the average FI values of individuals did not differ throughout the seasons, between habitats, and between maturity groups was tested using a three‐way ANOVA.

Diet composition was assessed using several indices reviewed by Hyslop (1980). Frequency of occurrence (FO%) was calculated to determine the prevalence of specific food items within samples. FO% was calculated as the percentage of stomachs containing a particular food category relative to the total number of stomachs examined. Furthermore, the Index of Relative Importance (IRI) was used to identify the most significant dietary contributors by integrating both the FO% and the abundance and volume of each food item (Pinkas et al. 1970). The IRI was calculated as follows: IRI = FO% × (N% + V%), where FO% is the frequency of occurrence of the food category, N% is the number of organisms in a specific food category relative to the total number of food organisms, and V% is the volume of organisms in a specific food category relative to the total volume of food organisms. IRI is then standardized to IRI% for comparison (Cortés 1997).

To assess ontogenetic variation in feeding habits, fish specimens were classified as mature or immature based on gonadal development and a threshold length‐at‐first maturity of 41.3 mm fork length. Gonadal development was used as the primary criterion, with individuals classified as mature if their gonads were developed and immature if not. To avoid misclassification caused by regressed ovaries, reproductive seasonality and fork length were also considered. Individuals with regressed gonads were classified as mature if their fork length exceeded the threshold of 41.3 mm, particularly outside the reproductive season. The threshold length was determined using logistic regression, which modeled maturity status as a binary response to fork length, identifying the length at which 50% of individuals were predicted to be mature (Chen and Paloheimo 1994).

To test for spatiotemporal and intraspecific variation in the feeding of *Gobio insuyanus*, permutational multivariate analysis of variance (PERMANOVA) was applied using the *adonis2* function from the ‘*vegan*’ package (Oksanen et al. 2024). Bray–Curtis dissimilarity was used as the distance measure, calculated after square root transformation of prey volume data to reduce the influence of dominant prey types and stabilize variance. This approach was performed to evaluate the effects of habitat, season, sex, and maturity stages, as well as their potential interactions, on the overall feeding patterns. To visualize differences in feeding composition across these factors, Canonical Analysis of Principal Coordinates (CAP) was employed instead of a multidimensional scaling (MDS), as it provides a constrained ordination approach that explicitly models group differences (Anderson and Willis 2003). Unlike nMDS, which is an unconstrained ordination that primarily seeks to optimize distances in a reduced‐dimensional space, CAP ensures that the ordination axes are directly aligned with predefined factors of interest, making it particularly suited for assessing how well feeding patterns are structured by habitat, season, sex, and maturity. Significant prey vectors (*p* ≤ 0.05) with Spearman correlations of ≥ 0.6 were fitted onto the CAP plots using the *envfit* function to represent the contributions of specific prey categories to the observed variation. To assess whether dietary variability differed across factors, we tested for homogeneity of multivariate dispersions using the *betadisper* function in the vegan package. This analysis quantified the dispersion (within‐group variability) of diet composition for habitat, season, sex, and maturity based on Bray‐Curtis distances. Significance was assessed via ANOVA, with a significant result indicating that individuals within a given factor level exhibited higher dietary variability than others. All multivariate analyses were based on the Bray–Curtis dissimilarity matrix of diet compositions. Statistical and multivariate analyses were performed using R (v2024.12.0) (R Core Team 2024) with the Rstudio interface (RStudio Team 2024).

The diversity profile of diet compositions was assessed using four indices: taxonomic richness (number of taxa), Simpson's Diversity Index (1‐D), Shannon Diversity Index (H), and Pielou's Evenness Index. Taxonomic richness represents the total number of prey taxa observed in the diet. Simpson's Diversity Index (1‐D) measures the probability that two individuals randomly selected from a sample belong to different taxa, calculated as:

$$1-D=1-\sum_{\left\{i=1\right\}}^{\left\{S\right\}}p_{i}^{2}$$

where *Pi* is the proportional abundance of the *i*‐th taxon, and *S* is the total number of taxa (Simpson 1949). The Shannon Diversity Index (*H*) quantifies diversity by incorporating both abundance and evenness of taxa (Spellerberg and Fedor 2003) and is calculated as:

$$H=-\sum_{\left\{i=1\right\}}^{\left\{S\right\}}\mathrm{Pi}.\ln\left(p_{i}\right)$$

Pielou's Evenness Index normalizes the Shannon Index to the range [0, 1], providing a measure of how evenly individuals are distributed among prey taxa, calculated as the exponential of the Shannon Index (*e* ^*H*^) divided by *S* (total number of taxa). These indices were computed using the *vegan* package in R (Oksanen et al. 2024).

To assess the feeding strategy, the modified Costello graphical method was employed (Amundsen et al. 1996). This method plots the prey‐specific abundance of each food category against its frequency of occurrence (FO%) on a two‐dimensional graph. Prey‐specific abundance (*P*_*i*_) was calculated using the formula: *P*_*i*_ = (∑S_*i*_ /∑T_*si*_) × 100, where *P*_*i*_ represents the prey‐specific abundance of food category *i*, *Si* is the volume of stomach content comprised of prey *i*, and *Tsi* is the total stomach content volume of only those individuals containing prey *i*. Using prey volume instead of prey number in this analysis provided a more comprehensive representation of the feeding strategy, particularly for food categories like algae and detritus, which cannot be accurately counted but contribute significantly to the diet composition.

Trophic level (TROPH) and its standard error (s.e.) were calculated using the stand‐alone ACCESS application of TROPHLAB (Pauly et al. 2000). The estimation was based on the following formula:

$${\text{TROPH}}_{i}=1+\sum_{j=1}^{G}{\mathrm{DC}}_{\mathrm{ij}}\;x\;\text{TROPH}j$$

Here, TROPH_*i*_ is defined as the trophic level of the consumer *i* (*G. insuyanus*), while TROPH*j* is the fractional trophic level of prey *j*, which is expressed as the proportion of prey *j* in the diet of consumer *i*.

## 5 Results

### 5.1 Sample Structure

The study sample consisted of 198 individuals from the Stream (48) and Spring (150) habitats. Fork lengths of the specimens ranged from 16.6 mm (an immature individual of undetermined sex) to 145.4 mm (a female). Among the identified specimens, the smallest female measured 23.5 mm, and the smallest male measured 36.7 mm. Fork lengths of immature specimens (*n* = 49) ranged from 16.6 mm to 41.3 mm. Fork lengths of females (*n* = 94) ranged from 23.5 mm to 38.1 mm, while it ranged from 36.7 mm to 62.4 mm in males (*n* = 94). The weight range of the specimens was from 0.05 g to 54.22 g, with the two smallest and largest individuals being female. Among the individuals with identified sexes, the weight of the smallest female was 1.34 g, and the smallest male weighed 0.56 g. The sample exhibited a female‐dominated sex structure, with 111 females and 38 males, resulting in a female‐to‐male ratio of 2.92:1. Immature individuals, comprising 20.8% of the stream sample (*n* = 10) and 26.0% of the spring habitat sample (*n* = 39) could not be sexed due to undeveloped gonads. Feeding ecology was assessed on a randomly selected subsample of 110 individuals (54 from the spring habitat and 56 from the stream habitat). Fish with damaged bodies or empty stomachs were excluded to ensure that only individuals with stomach contents that could be analyzed were examined. In addition, the subsample was selected to include representatives of all size groups to account for potential ontogenetic dietary variation.

### 5.2 Feeding Intensity

**FIGURE 2:**
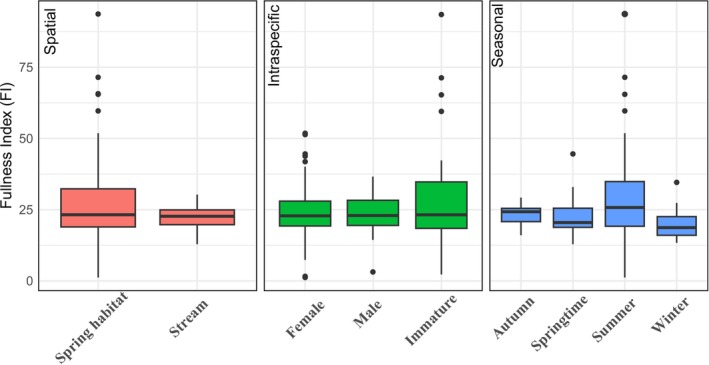
Boxplots of Fullness Index (FI) of *Gobio insuyanus* across spatial (left), intraspecific (middle), and seasonal (right) factors.

The fullness index (FI) varied across the factors examined, namely sex (including maturity), habitat, and season (Figure 2). FI was highest for fish sampled during the summer season and in spring habitats. Among the factors tested, habitat had a marginally significant effect on FI (ANOVA, = 0.047), suggesting that FI values tended to be higher in spring habitat compared to the stream. Although differences were observed between sexes and among seasons, these factors were not statistically significant and no significant interactions between factors were detected.

### 5.3 Overall Diet Description

A total of 21 prey categories were identified in the diet of *Gobio insuyanus* (Table 1). The diet primarily comprised detritus (organic debris and unidentifiable microscopic fragments) and a variety of invertebrates, dominated by insect larvae (mostly Diptera), Gammarids, and ostracods, with occasional contributions from other minor taxa (Table 1). Ostracods were the most abundant prey category regarding the percent numbers (N%). The only food item that was found throughout the year and in most of the individuals (87.30 FO%) was detritus. Detritus also had the highest contribution to the diet composition by volume (37.18%).

**TABLE 1:** *N*%, *V*%, FO% and IRI% values for stream habitat, spring habitat and overall combined habitats. The diversity indices and fractional trophic levels of the same factors in *Gobio insuyanus* are also provided.

Habitat type >	Stream habitat				Spring habitat				Overall			
Prey item	*N*%	*V*%	FO%	IRI%	*N*%	*V*%	FO%	IRI%	*N*%	*V*%	FO%	IRI%
Insecta (adult)												
Diptera	2.53	0.76	15.39	0.84	0.64	0.11	6.56	0.09	1.90	0.46	11.11	0.51
Corixidae	0.08	0.06	1.54	0.00	0.16	0.06	1.64	0.01	0.11	0.06	1.59	0.01
Curculionoidea	0.16	0.47	3.08	0.03	0.64	1.06	6.56	0.20	0.32	0.75	4.76	0.10
Insecta (larva)												
Diptera	30.70	23.38	66.15	59.31	5.25	2.26	22.95	3.16	22.27	13.51	45.24	31.67
Odonata	0.63	3.97	9.23	0.71	1.59	5.67	11.48	1.53	0.95	4.76	10.32	1.15
Plecoptera	1.66	5.56	16.92	2.03	3.82	7.25	16.39	3.32	2.38	6.35	16.67	2.85
Zooplankton												
*Chydorus sphaericus*	0.16	0.00	1.54	0.00	37.52	0.58	9.84	6.87	12.56	0.27	5.56	1.40
Naupli	0.16	0.00	3.08	0.01	0.00	0.00	0.00	0.00	0.11	0.00	1.59	0.00
Cyclopoida	5.53	0.13	20.00	1.88	0.32	0.00	1.64	0.01	3.80	0.07	11.11	0.84
Ostracoda	41.67	5.14	26.15	14.89	17.81	1.26	36.07	11.28	33.77	3.33	30.95	18.21
Other invertebrates												
Amphipoda	2.29	5.71	10.77	1.43	0.48	0.67	3.28	0.07	1.69	3.36	7.14	0.71
Gammaridae	7.18	17.91	43.08	17.92	27.98	39.51	59.02	72.99	14.09	28.00	50.79	41.83
Bivalvia	4.34	0.12	9.23	0.68	0.80	0.01	4.92	0.07	3.17	0.07	7.14	0.45
Gastropoda	0.16	0.04	3.08	0.01	0.64	0.10	6.56	0.09	0.32	0.07	4.76	0.04
Acariformes												
Terrestrial type	0.00	0.00	0.00	0.00	0.48	0.04	1.64	0.02	0.16	0.02	0.79	0.00
Aquatic type	1.11	0.01	7.69	0.14	0.16	0.00	1.64	0.01	0.79	0.00	4.76	0.07
Nematoda												
Short type	1.42	0.00	4.62	0.11	0.00	0.00	0.00	0.00	0.95	0.00	2.38	0.04
Long type	0.16	0.03	3.08	0.01	0.95	0.10	4.92	0.10	0.42	0.06	3.97	0.04
Vertebrates												
Fish larvae	0.08	0.63	1.54	0.02	0.64	2.87	3.28	0.21	0.26	1.68	2.38	0.09
Detritus	NA	36.09	86.15	NA	NA	38.43	88.53	NA	NA	37.18	87.30	NA
**Diversity profile**	**Stream habitat**				**Spring habitat**				**Overall**			
Taxa number	17				18				21			
Simpson 1‐D	0.76				0.56				0.75			
Shannon H	1.72				1.31				1.72			
Evenness_e^H/S	0.33				0.21				0.27			
Trophic Level (without detritus/with detritus)	3.27 ± 0.22/2.81 ± 0.14				3.32 ± 0.35/2.82 ± 0.21				3.30 ± 0.28/2.81 ± 0.18			

### 5.4 Spatiotemporal and Intraspecific Variation in Feeding

**FIGURE 3:**
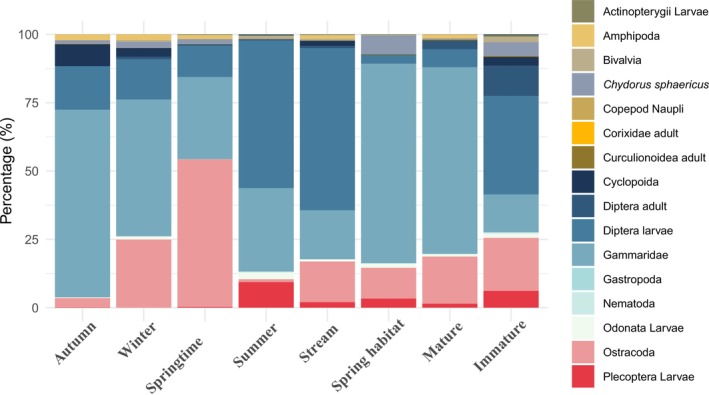
Index of Relative Importance (IRI%) of prey categories across seasons, habitats, and maturity stages in *Gobio insuyanus*.

**FIGURE 4:**
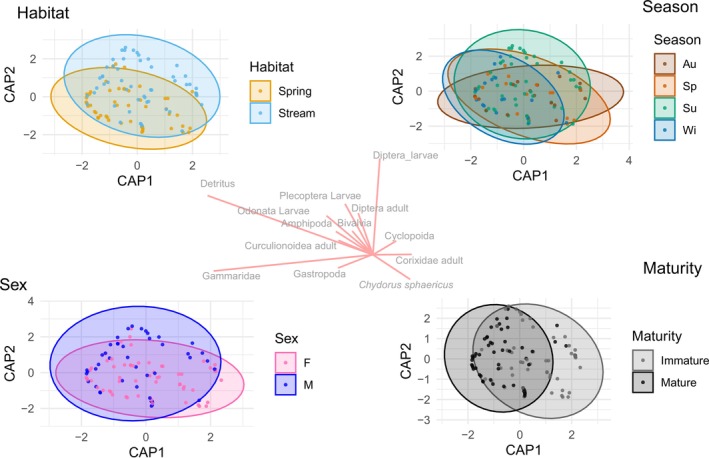
Canonical Analysis of Principal Coordinates (CAP) ordination plots showing the variation in diet composition of *Gobio insuyanus* across habitat (top‐left), season (top‐right), sex (bottom‐left), and maturity (bottom‐right). Each point represents an individual fish, with ellipses indicating the 95% confidence region for each factor level. Prey category vectors (center) represent significant contributors to dietary variation based on Spearman correlations (*p* ≤ 0.05, *r* ≥ 0.6). Au, autumn; F, female; M, male; Sp, springtime; Su, summer; Wi, winter.

The frequency, volume, and number of contributions made by food categories in each habitat and for each maturity stage are provided in Table 1 and Table 2, respectively. A summary illustration of the IRI% values across the studied factors is also provided in Figure 3. In the stream habitat, the three most significant prey items (based on IRI%) were identified as Diptera larvae (59.31%), Gammaridae (17.92%), and Ostracoda (14.89%). In contrast, the spring habitat exhibited a diet dominated by Gammaridae (72.99), followed by Ostracoda (11.28) and *Chydorus sphaericus* (6.87). PERMANOVA analysis resulted in significant variations in diet composition across habitats (spring vs. stream) (*F* = 9.06, *p* = 0.001), seasons (*F* = 2.17, *p* = 0.010), and maturity classes (mature vs. immature) (*F* = 7.28, *p* = 0.001), while sex had no significant effect (*F* = 0.93, *p* = 0.452) (Table 3). Detritus was excluded from this analysis (Table 3). However, including detritus as a prey category resulted in largely consistent results, although the seasonal variation became marginally significant (*F* = 1.59, *p* = 0.063). For overall CAP analysis, CAP1 (54.25%) and CAP2 (30.13%) accounted for most of the constrained variation, with habitat, season, and maturity contributing significantly to dietary separation, while sex showed no clear pattern (Figure 4). Prey categories contributing most to dietary variation included Gammaridae (CAP1: −1.75), Diptera larvae (CAP2: 1.38), and Detritus (CAP1: −1.52, CAP2: 0.89). Multivariate dispersion analysis revealed that only maturity significantly influenced dietary variability (*F* = 14.31, *p* = 0.00025), with immature individuals exhibiting more consistent dietary patterns compared to mature individuals. In contrast, habitat (*F* = 0.03, *p* = 0.864), season (*F* = 1.19, *p* = 0.318), and sex (*F* = 0.79, *p* = 0.377) did not show significant differences in dispersion, suggesting that while dietary composition varied across these factors, individual‐level variability remained similar within each group.

**TABLE 2:** *N*%, *V*% andFO% values for seasons and ontogenetic stages. The diversity indices and fractional trophic levels of the same factors in *Gobio insuyanus* are also provided.

Habitat type >	Summer			Autumn			Winter			Spring			Mature			Immature		
Prey item	*N*%	*V*%	FO%	*N*%	*V*%	FO%	*N*%	*V*%	FO%	*N*%	*V*%	FO%	*N*%	*V*%	FO%	*N*%	*V*%	FO%
Insecta (adult)																		
Diptera	0.00	0.00	0.00	3.70	1.32	11.11	0.81	0.37	21.74	1.72	0.25	11.29	17.37	0.44	10.91	29.63	0.51	14.55
Corixidae	0.00	0.00	0.00	0.00	0.00	0.00	0.16	0.17	4.35	0.17	0.06	1.61	0.35	0.04	1.82	0.26	0.11	1.82
Curculionoidea	0.79	1.14	4.35	0.18	0.62	5.56	0.16	0.72	4.35	0.52	0.72	4.84	2.19	0.69	7.27	2.65	0.90	3.64
Insecta (larva)																		
Diptera	17.32	6.49	43.48	15.67	14.13	33.33	7.09	8.21	43.48	46.03	16.50	50.00	0.09	8.74	49.09	0.13	26.09	54.55
Odonata	0.79	2.44	4.35	0.53	3.94	16.67	0.00	0.00	0.00	2.41	7.15	14.52	0.09	4.02	14.55	0.00	6.74	9.09
Plecoptera	0.79	1.30	4.35	0.00	0.00	0.00	0.32	1.65	8.70	7.24	11.44	29.03	0.96	4.87	16.36	0.93	10.27	21.82
Zooplankton																		
*Chydorus sphaericus*	9.45	0.13	8.70	25.70	0.84	5.56	11.59	0.48	8.70	1.38	0.02	3.23	6.84	0.12	3.64	21.16	0.67	9.09
Naupli	0.79	0.00	4.35	0.18	0.00	5.56	0.00	0.00	0.00	0.00	0.00	0.00	0.00	0.00	0.00	0.26	0.00	3.64
Cyclopoida	19.69	0.23	26.09	7.57	0.21	27.78	0.00	0.00	0.00	0.69	0.01	4.84	1.75	0.03	7.27	6.88	0.19	18.18
Ostracoda	8.66	0.52	30.44	31.16	4.50	66.67	69.08	13.05	47.83	3.97	0.24	14.52	42.80	3.49	29.10	20.11	2.89	34.55
Other invertebrates																		
Amphipoda	4.72	5.80	13.04	2.11	6.24	16.67	2.09	7.94	8.70	0.17	0.20	1.61	2.63	4.34	12.73	0.26	0.76	3.64
Gammaridae	35.43	43.47	56.52	10.21	30.16	83.33	7.57	28.72	47.83	20.17	23.69	40.32	20.70	34.14	81.82	4.10	11.83	34.55
Bivalvia	0.79	0.01	4.35	2.64	0.08	16.67	0.00	0.00	0.00	7.59	0.10	8.07	0.88	0.02	5.45	6.61	0.21	10.91
Gastropoda	0.00	0.00	0.00	0.35	0.12	11.11	0.32	0.14	8.70	0.35	0.05	3.23	0.44	0.08	9.09	0.13	0.04	1.82
Acariformes																		
Terrestrial type	0.00	0.00	0.00	0.00	0.00	0.00	0.48	0.10	4.35	0.00	0.00	0.00	0.26	0.02	1.82	0.00	0.00	0.00
Aquatic type	0.00	0.00	0.00	0.00	0.00	0.00	0.16	0.00	4.35	2.41	0.01	8.07	0.18	0.00	3.64	1.72	0.01	7.27
Nematoda																		
Short type	0.00	0.00	0.00	0.00	0.00	0.00	0.00	0.00	0.00	3.10	0.00	4.84	0.18	0.00	1.82	2.12	0.00	3.64
Long type	0.79	0.07	4.35	0.00	0.00	0.00	0.00	0.00	0.00	1.21	0.10	6.45	0.00	0.00	0.00	1.06	0.22	9.09
Vertebrates																		
Fish larvae	0.00	0.00	0.00	0.00	0.00	0.00	0.16	1.96	4.35	0.69	2.59	3.23	2.19	0.46	1.82	1.46	4.88	3.64
Detritus	NA	38.41	91.30	NA	37.84	100.0	NA	36.49	86.96	NA	36.89	82.26	0.09	38.51	100.0	0.53	33.68	NA
**Diversity profile**	**Summer**			**Autumn**			**Winter**			**Spring**			**Mature**			**Immature**		
Taxa number	16			12			12			14			17			17		
Simpson 1‐D	0.74			0.48			0.69			0.72			0.65			0.77		
Shannon H	1.55			1.07			1.53			1.59			1.50			1.79		
Evenness_e^H/S	0.30			0.24			0.38			0.35			0.26			0.35		
Trophic Level (without detritus/with detritus)	3.29 ± 0.24/2.81 ± 0.15			3.27 ± 0.38/2.78 ± 0.23			3.27 ± 0.28/2.79 ± 0.18			3.35 ± 0.28/2.86 ± 0.18			3.28 ± 0.31/2.79 ± 0.19			3.32 ± 0.21/2.88 ± 0.14		

**TABLE 3:** The main test results of PERMANOVA on the overall, seasonal, spatial, and ontogenetic differences in diet composition in *Gobio insuyanus* (Bold values indicates statistical significance at the *P* = 0.05).

Source	df	SS	MS	Pseudo‐*F*	*P* (perm)
Habitat (Ha)	1	19,849	19,849	9.0628	**0.001**
Season (Sea)	3	14,260	4753.5	2.1703	**0.010**
Sex	1	2039.4	2039.4	0.93117	0.452
Maturity (Ma)	1	15,943	15,943	7.2795	**0.001**
Ha × Sea	3	11,427	3808.9	1.7391	0.051
Ha × Sex	1	3971.9	3971.9	1.8135	0.076
Ha × Ma	1	4013.9	4013.9	1.8326	0.116
Sea × Sex	3	8813.6	2937.9	1.3414	0.173
Sea × Ma	3	5446.1	1815.4	0.82886	0.660
Sex × Ma	1	1972.9	1972.9	0.90078	0.476
Ha × Sea × Sex	3	5405.6	1801.9	0.8227	0.649
Ha × Sea × Ma	3	11,905	3968.4	1.8119	**0.038**
Ha × Sex × Ma	1	2871	2871	1.3108	0.248
Sea × Sex × Ma	2	2794.1	1397.1	0.63787	0.752

**FIGURE 5:**
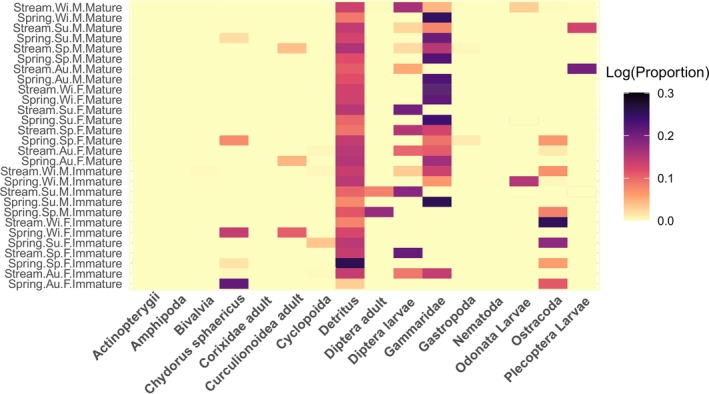
Heatmap showing the log‐transformed volumetric proportions of prey categories in *Gobio insuyanus* across different factor combinations. Rows represent unique combinations of habitat (spring, stream), season (Au, autumn; su, summer; sp, springtime; Wi, winter), sex (F, female; M, male), and maturity (Mature, immature). Columns correspond to different prey categories.

The heatmap of prey proportions (log‐transformed % volumes) demonstrated the combined effects of habitat, season, and ontogeny on dietary variation in the diet of *Gobio insuyanus* (Figure 5). Detritus consistently dominates in most combinations, serving as a major dietary component. Mature individuals exhibited a tendency to consume more Gammarids and Plecoptera larvae, while immature fish relied more heavily on smaller prey items such as *Chydorus sphaericus* and ostracods. Habitat differences also played a significant role in shaping diet composition, with ostracods being more prominent in the spring habitat, while Diptera larvae are more dominant in the stream habitat. The variation of IRI% across the studied factors (Figure 3) largely aligns with the observed patterns in the prey volume analysis, confirming the key contribution of Gammaridae and Diptera larvae as dietary components in multiple factor combinations. However, there are some discrepancies. For example, ostracods were prominent in spring habitats and among immature individuals in both datasets, but the IRI% data suggest that they are more significant in spring season samples (54.0%) than in terms of relative volume. Similarly, Diptera larvae are significant prey in stream habitat (59.3%), though they are less significant in the volume data. The exclusion of detritus from the IRI% analysis due to the absence of its abundance data (N%) is also notable, as its dominance in the volume data reveals its role as a dietary component.

### 5.5 Feeding Ecology

The diversity indices revealed higher levels of diet diversity and evenness in the stream habitat compared to the spring habitat. Autumn showed the lowest diversity indices among all seasons. The ontogenetic analysis indicated that mature individuals had lower diversity and evenness compared to immature individuals. Trophic level estimates were calculated with and without detritus. Trophic levels without detritus indicated an omnivorous diet with a preference for animal prey, while the levels with detritus suggested omnivory with a preference for plant organisms. The highest trophic level was obtained in the spring season.

**FIGURE 6:**
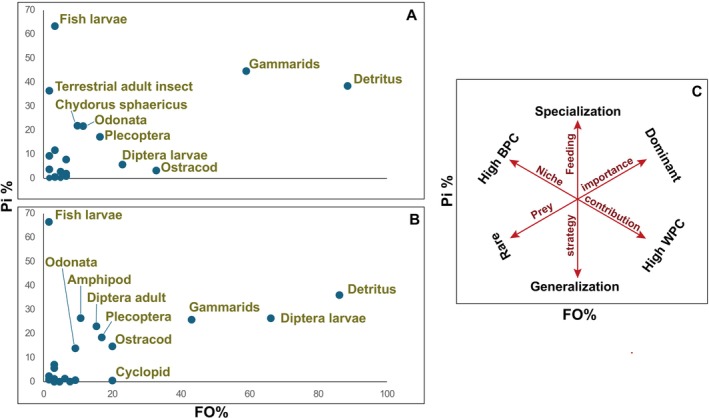
Prey importance plots using the modified Costello method for *Gobio insuyanus* in spring habitat (A) and stream habitat (B). The *x*‐axis represents frequency of occurrence (FO%), and the *y*‐axis shows prey‐specific abundance (*P* *i*%). (C) Explanatory diagram illustrating feeding strategies: Generalization, specialization, and dominance. BPC, between‐phenotype component; FO%, frequency of occurrence; *P* *i*%, prey‐specific abundance; WPC, within‐phenotype component.

According to the modified Costello graphical method, detritus dominates the diet in both habitats, exhibiting the highest prey‐specific abundance and frequency of occurrence (Figure 6). Gammaridae are also prominent in both habitats, while in the stream habitat, Diptera larvae are another key prey item in the population. In both habitats, there were individuals that fed occasionally but exclusively on fish larva of unidentified species (Figure 6).

## 6 Discussion

This study presents the first comprehensive analysis of the feeding ecology of *Gobio insuyanus*, a critically endangered gudgeon endemic to the Insuyu spring‐stream system in Central Anatolia (Turkey). The overall results clearly reveal that the species is omnivorous, with significant variation in its diet composition and feeding strategies across habitats, seasons, and maturity classes, suggesting that it exhibits adaptability in its feeding strategies in response to environmental factors and ontogenetic changes.

The consistent presence of detritus as the dominant category by volume in the diet of *Gobio insuyanus* reflects its benthic foraging behavior. As a bottom‐dwelling fish, *G. insuyanus* is likely to encounter and ingest detritus while foraging on the substrate. This is also consistent with findings from other gudgeon species, such as *Gobio gobio* (Kennedy and Fitzmaurice 1972) and *Gobio bulgaricus* (Saç and Özuluğ 2020), which also showed a significant reliance on benthic feeding, particularly on detritus and small invertebrates. The morphology of gudgeons, including their inferior mouth position and well‐developed sensory barbels, is adapted for feeding on benthic organisms and detrital matter. Al‐Hussaini (1949) also suggested that the protrusible mouth and barbels, together with specialized taste buds and mucus‐secreting cells throughout the alimentary tract of *Gobio* species, enhance their ability to detect and process food in benthic habitats, which supports the observed detritivory. The high frequency of occurrence (87.3%) and volumetric contribution (37.18%) of detritus further suggest that it may serve as a significant source of nutrition, especially when preferred prey items are less abundant. A study on *Gobio lozanoi* in the Iberian Peninsula also highlighted the importance of detritus in the diet of gudgeons, particularly in environments with limited prey availability such as in fluctuating environments (Oscoz et al. 2006). These results are in line with those of many other cypriniforms (Lobón‐Cerviá and Rincón 1994; McNeely 1987). For example, the red roach ( *Rutilus arcasii* ) has indicated similar shifts to detritus when higher‐quality food is limited, suggesting that this is a rational tactic to maintain energy intake without significant life history costs (Lobón‐Cerviá and Rincón 1994). However, this reliance on detritus, although expected for a substrate‐oriented species, has wider implications for interpreting the trophic level and ecological role of the species. Detritus, often considered an ambiguous food category, includes decomposed organic matter, fungi, bacterial components, and even inorganic remains (Bowen 1987). These elements can provide supplementary food, particularly during periods of low prey abundance, but their uncertain composition limits trophic assessment. When detritus was included in this study, the trophic level suggested omnivory with a preference for plant material, as detritus contributed similarly to plant material in its coefficient weight. However, excluding detritus provided a clearer understanding of the trophic position by demonstrating the reliance of the fish on animal prey such as *Gammarus* and diptera larvae. This double calculation shows the need for refinement of trophic assessment methods, particularly in freshwater systems where the role of detritus may obscure effective trophic levels due to its trophic coefficient, which may need to be reassessed. As noted in a previous work, functional trophic groups developed for marine fishes may not adequately capture the finer details of freshwater feeding ecology, as no specific freshwater categorization currently exists (Yoğurtçuoğlu et al. 2020).

Gammarids constituted another key dietary component, being particularly abundant in the spring habitat, where stable environmental conditions likely promoted high densities. Similar findings were observed in *G. bulgaricus*, where dipteran larvae (96.5% IRI) were the dominant dietary component, followed by crustaceans such as Gammaridae, suggesting a preference for energetically rich benthic prey (Saç and Özuluğ 2020). Although environmental prey abundance was not directly quantified in this study, field observations noted the common and abundant presence of *Gammarus* in the spring habitat, which is also true and consistent for ostracods, a finding that confirms the results and supports foraging theory, which emphasizes prey availability as a key determinant of feeding habits (Langerhans et al. 2021; Townsend and Winfield 1985). This finding is consistent with observations from other studies, which indicate that amphipods and ostracods thrive in benthic zones characterized by stable hydrological conditions. Furthermore, selective predation studies on *Gobio gobio* have confirmed that gammarids are actively selected as prey due to their high energy content and ease of capture in benthic environments (Worischka et al. 2015).

The observed differences in feeding intensity and diet diversity between the spring and stream habitats can be attributed to contrasting environmental characteristics. Spring habitat exhibits higher feeding intensity, particularly during the summer, likely due to a combination of factors: a stable thermal regime with temperatures consistently ranging from 13.5°C to 15.5°C, a more complex habitat structure with a coarser substrate, and lower turbidity. These conditions likely create a more favorable environment for many prey organisms, resulting in higher prey availability (Jönsson et al. 2013; Turesson and Brönmark 2007). Reduced turbidity can enhance visibility, potentially increasing foraging efficiency for a visually‐oriented fish. Worischka et al. (2015) demonstrated that gudgeon feeding patterns are highly influenced by microhabitat stability, with prey availability and water clarity playing crucial roles in their foraging efficiency. The higher proportion of immature individuals in the spring habitat may also contribute to the more variable FI values observed there, as ontogenetic dietary shifts can lead to greater feeding flexibility. Considering the life history traits studied in other gudgeons, the increased feeding intensity in summer may also coincide with the peak reproductive period, when energy demands are higher, as observed in other *Gobio* species (Saç and Özuluğ 2020). In contrast, the stream habitat, characterized by fluctuating water temperatures and a finer substrate, exhibits higher diet diversity and evenness, suggesting potentially greater variability in environmental conditions and a wider range of supported prey taxa. While the stability in spring habitat seems to favor certain prey species (e.g., Gammarids), leading to a less diverse but potentially more abundant food source, variability in stream habitat may promote a broader, yet possibly less concentrated, prey base. However, the lower diet diversity observed across both habitats in autumn likely reflects a seasonal decline in overall prey availability, irrespective of habitat‐specific conditions. Research by Blackman et al. shows that invertebrate richness is significantly affected by seasonal changes, particularly by the emergence of nonaquatic adult life stages of several macroinvertebrate genera in late spring and summer. This emergence results in a reduction of these organisms in aquatic environments as they transition to terrestrial habitats (Blackman et al. 2022). Similarly, Carraro et al. (2023) observed a decrease in invertebrate counts in autumn compared to spring and summer, indicating a lower abundance of invertebrates during this season. Furthermore, Kreiling et al. noted that the community structure of invertebrates varies significantly between seasons, with certain taxa being more abundant in winter and summer, while autumn shows a marked decrease in diversity (Kreiling et al. 2020). In addition to feeding intensity and diet diversity, the observed spatiotemporal variations in overall diet composition are likely driven by a combination of factors including habitat‐specific energy demands and potentially competitive interactions. The seasonal shift in prey consumption patterns likely reflects changes in the abundance and diversity of available prey in both habitats. For instance, the higher consumption of Diptera larvae in the stream during certain seasons may correspond to increased emergence or larval abundance of these insects. For instance, a study on the seasonal abundance of aquatic Diptera in two oligohaline tidal marshes in Mississippi found distinct seasonal patterns in Diptera populations, which in turn affected their availability to predators (LaSalle and Dale Bishop 1987). Furthermore, the direct exposure of the stream habitat to agricultural nutrient loads may provide a suitable environment for the development of Dipteran larvae, consequently contributing to their high population numbers. Indeed, the findings of Powell et al. (2024) indicated that increases in Diptera abundance over time were greater in areas with higher agricultural intensity, suggesting a positive correlation between agricultural activities and Dipteran populations.

No significant dietary differences were found between sexes, suggesting that males and females share similar foraging strategies and trophic niches. However, juveniles were observed to consume primarily smaller prey items, such as ostracods and *Chydorus sphaericus* , while adults exhibited a shift towards larger prey, including Gammarids and Plecoptera larvae. This shift, which parallels findings in *G. gobio* (Kennedy and Fitzmaurice 1972) and *G. lozanoi* (Oscoz et al. 2006), is likely to reflect both the increased gape size and foraging ability associated with growth and the increased energy requirements of reproduction in mature individuals. As fish grow, they become capable of capturing and consuming larger prey while simultaneously requiring more energy, particularly during the reproductive period. This may necessitate a shift to higher‐energy prey items. This ontogenetic dietary shift is consistent with the broader ecological role of ontogenetic niche shifts as fundamental drivers of ecosystem coupling (Sánchez‐Hernández, 2024). Multivariate dispersion analysis further supports the importance of ontogenetic dietary shifts, as maturity was the only factor that significantly influenced dietary variability (*p* = 0.00025). While mature individuals exhibited greater dietary dispersion, this does not imply a broader dietary niche at the population level. Rather, it suggests higher individual variability in prey selection, with some individuals specializing in energetically rich prey like Gammarids, while others incorporate a more diverse prey types. This aligns with the observed lower dietary evenness in mature individuals, indicating that while their diet may be compositionally different, individual dietary choices vary more widely than in juveniles, who rely consistently on smaller, more uniform prey. The absence of significant dispersion differences across habitat and season, despite their effect on dietary composition, suggests that while the examined factors shape diet composition, individuals within each group exhibit similar levels of dietary consistency. This ontogenetic shift appears to also cause a trend towards dietary specialization. For example, in the predatory fish *Ptychochromis elapoides*, larger individuals exhibit a preference for energetically more profitable prey, resulting in an ontogenetic diet shift (Choi and Suk 2012). Additionally, a global synthesis on ontogenetic dietary shifts in fish indicates that such shifts can enhance individual growth and lifetime reproductive output, which often leads to increased dietary specialization as fish mature (Sánchez‐Hernández et al. 2019). The lower dietary diversity and evenness observed in mature individuals suggest a narrowing of their dietary niche. This narrowing of the dietary niche may be driven by increased efficiency in capturing preferred prey items and potentially by increased intraspecific competition among larger individuals, consistent with optimal foraging theory, which predicts specialization on the most profitable prey when competition is high (Sánchez‐Hernández et al. 2012).

While this study provides valuable insights into the feeding ecology of *G. insuyanus* through stomach content analysis, we acknowledge the limitation of not directly quantifying prey abundance in the environment. This omission may introduce uncertainty into the interpretation of dietary variation and prey selection patterns. However, the observed dietary shifts across habitats, seasons, and maturity classes suggest that prey availability significantly influences feeding behavior. These findings are consistent with ecological theory, which suggests that prey abundance and environmental conditions play a critical role in shaping fish diets (Pouilly et al. 2006; Wang et al. 2019). Dietary overlap studies, such as those by Magalhães (1993), also suggest that in dynamic stream systems, interspecific competition and seasonal fluctuations in prey abundance can significantly shape feeding behavior. While stomach content analysis provides a snapshot of recent feeding intensity, future studies could benefit from incorporating stable isotope analysis to infer long‐term dietary assimilation and trophic relationships (Post 2002). Additionally, incorporating data on fish habitat use and movement patterns, potentially through methods like mark‐recapture or acoustic telemetry, would enhance our understanding of observed dietary variations. Despite the inherent limitations of this study, its design integrating several factors provides a valuable basis for exploring the feeding ecology of this imperiled species.

The Costello method revealed a certain degree of specialization within the population. While Gammarids were a key prey item in both habitats, their relative abundance in the spring, together with the higher feeding intensity observed there, suggests that they represent a particularly important resource in this stable environment. Conversely, the importance of Diptera larvae in the stream habitat indicates a potential opportunistic feeding on seasonally abundant prey in a more variable environment. The presence of individuals in both habitats that exclusively consume fish larvae indicates limited piscivory, a phenomenon that has not previously been reported for any *Gobio* species.

## 7 Practical Applications, Conservation Implications, and a Final Word

The findings of this study carry significant implications for the conservation of *G. insuyanus*. The observed habitat‐specific dietary differences suggest that it is essential to maintain the integrity of both the spring and stream habitats to ensure the availability of a diverse prey base for the species. This includes addressing the key threat of habitat loss driven by groundwater overuse. Water management strategies must prioritize the conservation of both habitats, acknowledging their distinct ecological functions. Furthermore, the species displays a degree of feeding plasticity, indicating a capacity to adapt to certain environmental changes. However, the dietary specialization exhibited by mature individuals may potentially render them more vulnerable to fluctuations in the availability of preferred prey items. Consequently, conservation efforts should prioritize the mitigation of threats to prey populations, including pollution, habitat degradation, and loss. The observed importance of the spring habitat for feeding, particularly during the summer months, suggests that this habitat may be critical for the species' survival, especially during periods of high energy demand associated with reproduction.

The information obtained in this study can be usefully applied to inform efforts directed towards habitat restoration. For instance, when considering the restoration of stream habitats, the substrate composition should be designed to support a diverse benthos assemblage. Moreover, the dietary information provided in this study could prove beneficial to captive breeding programmes. Captive diets should be formulated to mirror natural diets, incorporating detritus and accounting for ontogenetic shifts in prey preferences. It is further recommended that smaller prey items be included in the diets of immature fish, and that larger prey items be included in the diets of mature individuals, thus mimicking the natural shift observed in the wild.

Finally, for this study, we sampled *G. insuyanus* from June 2018 to September 2019, providing a baseline assessment of its feeding ecology. Since then, we have continued to monitor the habitat of the species with annual but irregular site visits until the submission of this paper (February 2025). Unfortunately, during these subsequent assessments, we have observed a dramatic decline in both spring and stream habitat, with significant habitat loss over time. Ongoing degradation due to groundwater overexploitation and habitat fragmentation now poses an even greater threat to the survival of the species. These findings demonstrate the critical need for immediate conservation action to mitigate further habitat loss and ensure the long‐term survival of *G. insuyanus* in its native restricted range.

## Author Contributions

**Julian E. Johnson:** data curation (lead), investigation (equal), writing – review and editing (supporting). **Baran Yoğurtçuoğlu:** conceptualization (equal), formal analysis (lead), methodology (lead), writing – original draft (lead), writing – review and editing (supporting). **Şerife Gülsün Kırankaya:** investigation (equal), methodology (supporting), writing – review and editing (supporting). **Fitnat Güler Ekmekçi:** conceptualization (equal), investigation (equal), writing – original draft (supporting), writing – review and editing (supporting).

## Conflicts of Interest

The authors declare no conflicts of interest.

## Data Availability

All data can be downloaded from the dryad repository at https://doi.org/10.5061/dryad.cz8w9gjft.

## References

[ece371156-bib-0001] Al‐Hussaini, A. H. 1949. “On the Functional Morphology of the Alimentary Tract of Some Fish in Relation to Differences in Their Feeding Habits: Anatomy and Histology.” Journal of Cell Science 3, no. 10: 109–139. 10.1242/jcs.s3-90.10.109. 18132292

[ece371156-bib-0002] Amundsen, P.‐A. , H.‐M. Gabler , and F. J. Staldvik . 1996. “A New Approach to Graphical Analysis of Feeding Strategy From Stomach Contents Data—Modification of the Costello (1990) Method.” Journal of Fish Biology 48, no. 4: 607–614. 10.1111/j.1095-8649.1996.tb01455.x.

[ece371156-bib-0003] Anderson, M. J. , and T. J. Willis . 2003. “Canonical Analysis of Principal Coordinates: A Useful Method of Constrained Ordination for Ecology.” Ecology 84, no. 2: 511–525. 10.1890/0012-9658(2003)084[0511:CAOPCA]2.0.CO;2.

[ece371156-bib-0004] Blackman, R. C. , H.‐C. Ho , J.‐C. Walser , and F. Altermatt . 2022. “Spatio‐Temporal Patterns of Multi‐Trophic Biodiversity and Food‐Web Characteristics Uncovered Across a River Catchment Using Environmental DNA.” Communications Biology 5, no. 1: 259. 10.1038/s42003-022-03216-z. 35322190 PMC8943070

[ece371156-bib-0005] Bowen, S. H. 1987. “Composition and Nutritional Value of Detritus.” In Detritus and Microbial Ecology in Aquaculture: Proceedings of the Conference on Detrital Systems for Aquaculture, 192–216. Microbial Ecology in Aquaculture.

[ece371156-bib-0006] Bozdağ, A. , and G. Göçmez . 2013. “Evaluation of Groundwater Quality in the Cihanbeyli Basin, Konya, Central Anatolia, Turkey.” Environmental Earth Sciences 69, no. 3: 921–937. 10.1007/s12665-012-1977-4.

[ece371156-bib-0007] Carraro, L. , R. C. Blackman , and F. Altermatt . 2023. “Modelling Environmental DNA Transport in Rivers Reveals Highly Resolved Spatio‐Temporal Biodiversity Patterns.” Scientific Reports 13, no. 1: 8854. 10.1038/s41598-023-35614-6. 37258598 PMC10232434

[ece371156-bib-0008] Chen, Y. , and J. E. Paloheimo . 1994. “Estimating Fish Length and Age at 50% Maturity Using a Logistic Type Model.” Aquatic Sciences 56, no. 3: 206–219. 10.1007/BF00879965.

[ece371156-bib-0009] Choi, S.‐H. , and H. Y. Suk . 2012. “The Mechanisms Leading to Ontogenetic Diet Shift in a Microcanivore, *Pterogobius elapoides* (Gobiidae).” Animal Cells and Systems 16, no. 4: 343–349. 10.1080/19768354.2012.667002.

[ece371156-bib-0010] Cortés, E. 1997. “A Critical Review of Methods of Studying Fish Feeding Based on Analysis of Stomach Contents: Application to Elasmobranch Fishes.” Canadian Journal of Fisheries and Aquatic Sciences 54, no. 3: 726–738.

[ece371156-bib-0011] Darwall, W. , S. Carrizo , C. Numa , V. Barrios , J. Freyhof , and K. Smith . 2015. Freshwater Key Biodiversity Areas in the Mediterranean Basin Hotspot. International Union for Conservation of Nature. 10.2305/IUCN.CH.2014.SSC-OP.52.en.

[ece371156-bib-0012] DKM . 2019. Sürdürülebilir Arazi Yönetimi ve İklim Dostu Tarım Projesi: “Biyolojik Çeşitlilik Envanteri ve Biyolojik Çeşitlilik Yönetim Planı”, 143. FAO.

[ece371156-bib-0013] Ergönül, M. , J. Breine , and S. Atasagun . 2019. “Length‐Weight Relationships of Two Threatened *Gobio* Species Endemic to Turkey: *Gobio* *insuyanus* Ladiges and *Gobio microlepidotus* Battalgil.” Fisheries & Aquatic Life 27, no. 3: 118–121. 10.2478/aopf-2019-0013.

[ece371156-bib-0014] Erk'akan, F. , D. Innal , and F. Özdemir . 2014. “Length–Weight Relationships for Five Cyprinid Species in Turkey.” Journal of Applied Ichthyology 30, no. 1: 212–213. 10.1111/jai.12356.

[ece371156-bib-0015] Freyhof, J. , B. Yoğurtçuoğlu , A. Jouladeh‐Roudbar , and C. Kaya . 2025. Handbook of Freshwater Fishes of West Asia. De Gruyter. https://www.degruyter.com/document/isbn/9783111678245/html.

[ece371156-bib-0016] Hellawell, J. , and R. Abel . 2006. “A Rapid Volumetric Method for the Analysis of the Food of Fishes.” Journal of Fish Biology 3: 29–37. 10.1111/j.1095-8649.1971.tb05903.x.

[ece371156-bib-0017] Hyslop, E. J. 1980. “Stomach Contents Analysis—A Review of Methods and Their Application.” Journal of Fish Biology 17, no. 4: 411–429. 10.1111/j.1095-8649.1980.tb02775.x.

[ece371156-bib-0018] Jönsson, M. , L. Ranåker , P. A. Nilsson , and C. Brönmark . 2013. “Foraging Efficiency and Prey Selectivity in a Visual Predator: Differential Effects of Turbid and Humic Water.” Canadian Journal of Fisheries and Aquatic Sciences 70, no. 12: 1685–1690. 10.1139/cjfas-2013-0150.

[ece371156-bib-0019] Kennedy, M. , and P. Fitzmaurice . 1972. “Some Aspects of the Biology of Gudgeon *Gobio gobio* (L.) in Irish Waters.” Journal of Fish Biology 4, no. 3: 425–440. 10.1111/j.1095-8649.1972.tb05690.x.

[ece371156-bib-0020] Korkmaz, M. , F. Mangıt , İ. Dumlupınar , et al. 2023. “Effects of Climate Change on the Habitat Suitability and Distribution of Endemic Freshwater Fish Species in Semi‐Arid Central Anatolian Ecoregion in Türkiye.” Water 15, no. 8: 1619. 10.3390/w15081619.

[ece371156-bib-0021] Kreiling, A.‐K. , E. J. O'Gorman , S. Pálsson , et al. 2020. “Seasonal Variation in the Invertebrate Community and Diet of a Top Fish Predator in a Thermally Stable Spring.” Hydrobiologia 848, no. 3: 531–545. 10.1007/s10750-020-04409-5.

[ece371156-bib-0022] Ladiges, W. 1960. “Süßwasserfische der Türkei, I. Teil Cyprinidae.” Mitteilungen Aus Dem Hamburgischen Zoologischen Museum Und Institut 58: 105–150.

[ece371156-bib-0023] Langerhans, R. B. , T. R. Goins , K. M. Stemp , R. Riesch , M. S. Araújo , and C. A. Layman . 2021. “Consuming Costly Prey: Optimal Foraging and the Role of Compensatory Growth.” Frontiers in Ecology and Evolution 8: 603387. 10.3389/fevo.2020.603387.

[ece371156-bib-0024] LaSalle, M. W. , and T. Dale Bishop . 1987. “Seasonal Abundance of Aquatic Diptera in Two Oligohaline Tidal Marshes in Mississippi.” Estuaries 10, no. 4: 303–315. 10.2307/1351888.

[ece371156-bib-0025] Lobón‐Cerviá, J. , and P. A. Rincón . 1994. “Trophic Ecology of Red Roach (*Rutilus arcasii*) in a Seasonal Stream; an Example of Detritivory as a Feeding Tactic.” Freshwater Biology 32, no. 1: 123–132. 10.1111/j.1365-2427.1994.tb00872.x.

[ece371156-bib-0026] Magalhães, M. F. 1993. “Feeding of an Iberian Stream Cyprinid Assemblage: Seasonality of Resource Use in a Highly Variable Environment.” Oecologia 96, no. 2: 253–260. 10.1007/BF00317739. 28313422

[ece371156-bib-0027] McNeely, D. L. 1987. “Niche Relations Within an Ozark Stream Cyprinid Assemblage.” Environmental Biology of Fishes 18, no. 3: 195–208. 10.1007/BF00000359.

[ece371156-bib-0028] Oksanen, J. , G. L. Simpson , F. G. Blanchet , et al. 2024. “Vegan: Community Ecology Package [Computer Software].” 10.32614/CRAN.package.vegan.

[ece371156-bib-0029] Oscoz, J. , P. M. Leunda , R. Miranda , and M. C. Escala . 2006. “Summer Feeding Relationships of the Co‐Occurring Phoxinus Phoxinus and *Gobio lozanoi* (Cyprinidae) in an Iberian River.” Folia Zoologica 55, no. 4: 418–432.

[ece371156-bib-0030] Özdemir, F. 2012. “Growth and Reproductive Biology of *Gobio* *g* *ymnostethus* (Ladiges, 1960) in Melendiz Stream, Anatolia, Turkey.” Journal of Animal and Veterinary Advances 11: 3452–3456. 10.3923/javaa.2012.3452.3456.

[ece371156-bib-0031] Özdemir, F. , and F. Erkakan . 2012. “Growth and Reproductive Properties of an Endemic Species, *Gobio hettitorum* Ladiges, 1960, in Yeşildere Stream, Karaman, Turkey.” Hacettepe Journal of Biology and Chemistry 40, no. 4: 457–468.

[ece371156-bib-0032] Pauly, D. , R. Froese , P. S. Sa‐a , M. L. Palomares , V. Christensen , and J. Rius . 2000. Trophlab Manual. ICLARM.

[ece371156-bib-0033] Pinkas, L. , M. Oliphant , and I. Iverson . 1970. Food Habits of Albacore, Bluefin Tuna, and Bonito in California Waters. Vol. 152, 105. Scripps Institution of Oceanography Library.

[ece371156-bib-0034] Post, D. M. 2002. “Using Stable Isotopes to Estimate Trophic Position: Models, Methods, and Assumptions.” Ecology 83, no. 3: 703–718. 10.2307/3071875.

[ece371156-bib-0035] Pouilly, M. , S. Barrera , and C. Rosales . 2006. “Changes of Taxonomic and Trophic Structure of Fish Assemblages Along an Environmental Gradient in the Upper Beni Watershed (Bolivia).” Journal of Fish Biology 68, no. 1: 137–156. 10.1111/j.0022-1112.2006.00883.x.

[ece371156-bib-0036] Powell, K. E. , D. Garrett , D. B. Roy , T. H. Oliver , M. Larrivée , and M. Bélisle . 2024. “Complex Temporal Trends in Biomass and Abundance of Diptera Communities Driven by the Impact of Agricultural Intensity.” Insect Conservation and Diversity 17, no. 6: 1072–1083. 10.1111/icad.12770.

[ece371156-bib-0037] R Core Team . 2024. R: A Language and Environment for Statistical Computing [Computer Software]. R Foundation for Statistical Computing. https://www.R‐project.org/.

[ece371156-bib-0038] Ricciardi, A. , and J. B. Rasmussen . 1999. “Extinction Rates of North American Freshwater Fauna.” Conservation Biology 13, no. 5: 1220–1222. 10.1046/j.1523-1739.1999.98380.x.

[ece371156-bib-0039] RStudio Team . 2024. RStudio: Integrated Development for R. RStudio, PBC (Version 2024.12.0). http://www.rstudio.com/.

[ece371156-bib-0040] Saç, G. , and M. Özuluğ . 2020. “Life History Pattern and Feeding Habits of *Gobio bulgaricus* (Drensky, 1926) (Pisces: Gobionidae) in an Endorheic Stream (Istranca Stream, Turkey).” Iranian Journal of Fisheries Sciences 19, no. 1: 248–261. 10.22092/ijfs.2019.118805.

[ece371156-bib-0041] Sala, O. E. , F. I. Stuart Chapin , and J. J. Armesto . 2000. “Global Biodiversity Scenarios for the Year 2100.” Science 287, no. 5459: 1770–1774. 10.1126/science.287.5459.1770. 10710299

[ece371156-bib-0042] Sánchez‐Hernández, J. 2024. “Climate‐Induced Shifts in Ontogenetic Niches Threaten Ecosystem Coupling.” Trends in Ecology & Evolution 40, no. 3: 224–227. 10.1016/j.tree.2024.11.018. 39690055

[ece371156-bib-0043] Sánchez‐Hernández, J. , A. D. Nunn , C. E. Adams , and P.‐A. Amundsen . 2019. “Causes and Consequences of Ontogenetic Dietary Shifts: A Global Synthesis Using Fish Models.” Biological Reviews of the Cambridge Philosophical Society 94, no. 2: 539–554. 10.1111/brv.12468. 30251433

[ece371156-bib-0044] Sánchez‐Hernández, J. J. M. , R. Vieira‐Lanero , and F. Cobo . 2012. “Ontogenetic Dietary Shifts in a Predatory Freshwater Fish Species: The Brown Trout as an Example of a Dynamic Fish Species.” In New Advances and Contributions to Fish Biology, 271. IntechOpen. 10.5772/54133.

[ece371156-bib-0045] Schindelin, J. , I. Arganda‐Carreras , E. Frise , et al. 2012. “Fiji: An Open‐Source Platform for Biological‐Image Analysis.” Nature Methods 9, no. 7: 676–682. 10.1038/nmeth.2019. 22743772 PMC3855844

[ece371156-bib-0046] Seabra, L. B. , N. L. Benone , and L. F. A. Montag . 2023. “Assessing the Effects of Multiple Land Uses on the Functional Beta Diversity of Stream Fishes in the Amazon Region.” Hydrobiologia 849, no. 20: 4515–4527. 10.1007/s10750-023-05284-z.

[ece371156-bib-0047] Şenyi̇ği̇t, G. , and R. E. Mazlum . 2022. “Türkiye İç Sularında Dağılım Gösteren 13 *Gobio* Türünün Boy‐Ağırlık İlişkisi.” Journal of Anatolian Environmental and Animal Sciences 7, no. 4: 472–478. 10.35229/jaes.1141576.

[ece371156-bib-0048] Simpson, E. H. 1949. “Measurement of Diversity.” Nature 163, no. 4148: 688. 10.1038/163688a0.

[ece371156-bib-0049] Smith, K. G. , V. Barrios , W. R. T. Darwall , and C. Numa . 2014. The Status and Distribution of Freshwater Biodiversity in the Eastern Mediterranean. IUCN. 10.2305/IUCN.CH.2014.01.en.

[ece371156-bib-0050] Spellerberg, I. F. , and P. J. Fedor . 2003. “A Tribute to Claude Shannon (1916–2001) and a Plea for More Rigorous Use of Species Richness, Species Diversity and the ‘Shannon–Wiener’ Index.” Global Ecology and Biogeography 12, no. 3: 177–179. 10.1046/j.1466-822X.2003.00015.x.

[ece371156-bib-0051] Strayer, D. L. , and D. Dudgeon . 2010. “Freshwater Biodiversity Conservation: Recent Progress and Future Challenges.” Journal of the North American Benthological Society 29, no. 1: 344–358. 10.1899/08-171.1.

[ece371156-bib-0052] Sun, J. , and D. Liu . 2003. “Geometric Models for Calculating Cell Biovolume and Surface Area for Phytoplankton.” Journal of Plankton Research 25, no. 11: 1331–1346. 10.1093/plankt/fbg096.

[ece371156-bib-0053] Tickner, D. , J. J. Opperman , R. Abell , et al. 2020. “Bending the Curve of Global Freshwater Biodiversity Loss: An Emergency Recovery Plan.” Bioscience 70, no. 4: 330–342. 10.1093/biosci/biaa002. 32284631 PMC7138689

[ece371156-bib-0054] Tockner, K. 2021. Freshwaters: Global Distribution, Biodiversity, Ecosystem Services, and Human Pressures, 489–501. Springer. 10.1007/978-3-030-60147-8_16.

[ece371156-bib-0055] Townsend, C. R. , and I. J. Winfield . 1985. “The Application of Optimal Foraging Theory to Feeding Behaviour in Fish.” In Fish Energetics: New Perspectives, edited by P. Tytler and P. Calow , 67–98. Springer Netherlands. 10.1007/978-94-011-7918-8_3.

[ece371156-bib-0056] Turesson, H. , and C. Brönmark . 2007. “Predator–Prey Encounter Rates in Freshwater Piscivores: Effects of Prey Density and Water Transparency.” Oecologia 153, no. 2: 281–290. 10.1007/s00442-007-0728-9. 17453254

[ece371156-bib-0057] Wang, S. , J.‐P. Tang , L.‐H. Su , et al. 2019. “Fish Feeding Groups, Food Selectivity, and Diet Shifts Associated With Environmental Factors and Prey Availability Along a Large Subtropical River, China.” Aquatic Sciences 81, no. 2: 31. 10.1007/s00027-019-0628-1.

[ece371156-bib-0058] Worischka, S. , S. I. Schmidt , C. Hellmann , and C. Winkelmann . 2015. “Selective Predation by Benthivorous Fish on Stream Macroinvertebrates—The Role of Prey Traits and Prey Abundance.” Limnologica 52: 41–50. 10.1016/j.limno.2015.03.004.

[ece371156-bib-0059] Yılmaz, G. , M. A. Çolak , İ. K. Özgencil , et al. 2021. “Decadal Changes in Size, Salinity, Waterbirds, and Fish in Lakes of the Konya Closed Basin, Turkey, Associated With Climate Change and Increasing Water Abstraction for Agriculture.” Inland Waters 11, no. 4: 538–555. 10.1080/20442041.2021.1924034.

[ece371156-bib-0060] Yoğurtçuoğlu, B. , F. G. Ekmekçi , and P. K. Karachle . 2020. “A Review and Assessment of Fish Trophic Levels in a Large Reservoir of Central Anatolia, Turkey.” Marine and Freshwater Research 72, no. 3: 311–320.

